# Cyclosporine Lipid Nanocapsules as Thermoresponsive Gel for Dry Eye Management: Promising Corneal Mucoadhesion, Biodistribution and Preclinical Efficacy in Rabbits

**DOI:** 10.3390/pharmaceutics13030360

**Published:** 2021-03-09

**Authors:** Lubna M. Eldesouky, Riham M. El-Moslemany, Alyaa A. Ramadan, Mahmoud H. Morsi, Nawal M. Khalafallah

**Affiliations:** 1 Department of Pharmaceutics, Faculty of Pharmacy, Alexandria University, Alexandria 21523, Egypt; lubna.eldesouky@alexu.edu.eg (L.M.E.); alyaa.ramadan@alexu.edu.eg (A.A.R.); nawal.khalafallah@alexu.edu.eg (N.M.K.); 2 Department of Ophthalmology, Faculty of Medicine, Alexandria University, Alexandria 21523, Egypt; morsimahmoud@yahoo.de

**Keywords:** cylclosporine A, lipid nanocapsules, thermoresponsive gel, corneal delivery, dry eye

## Abstract

An ophthalmic cyclosporine (CsA) formulation based on Lipid nanocapsules (LNC) was developed for dry eye management, aiming to provide targeting to ocular tissues with long-term drug levels and maximum tolerability. CsA-LNC were of small particle size (41.9 ± 4.0 nm), narrow size distribution (PdI ≤ 0.1), and high entrapment efficiency (above 98%). Chitosan (C) was added to impart positive charge. CsA-LNC were prepared as in-situ gels using poloxamer 407 (P). Ex vivo mucoadhesive strength was evaluated using bovine cornea, while in vivo corneal biodistribution (using fluorescent DiI), efficacy in dry eye using Schirmer tear test (STT), and ocular irritation using Draize test were studied in rabbits compared to marketed ophthalmic CsA nanoemulsion (CsA-NE) and CsA in castor oil. LNC incorporation in in-situ gels resulted in an increase in mucoadhesion, and stronger fluorescence in corneal layers seen by confocal microscopy, compared to the other tested formulations. Rate of recovery (days required to restore corneal baseline hydration level) assessed over 10 days, showed that CsA-LNC formulations produced complete recovery by day 7 comparable to CsA-NE. No Ocular irritation was observed by visual and histopathological examination. Based on data generated, CsA-LNC-CP in-situ gel proved to be a promising effective nonirritant CsA ophthalmic formulation for dry eye management.

## 1. Introduction

The present study was motivated by the need for effective affordable cyclosporine A (CsA) eye drops for the management of Dry Eye disease (DED). According to the Tear Film and Ocular surface Society Second Dry Eye Workshop (TFOS DEWS II) [1], prevalence of DED ranged between 5–50% reaching up to 75% in some populations, higher in Asian than in Caucasian populations. Linear increase in prevalence is evident with increase in age, with women more prone to DED than men [2]. If untreated, patients with dry eye may suffer potentially blinding infections, such as bacterial keratitis [3]. CsA is the anti-inflammatory of choice for treatment of DED, particularly severe cases; it is suitable for long term use without adverse effects commonly associated with corticosteroids [4]. CsA is currently available in Egypt for delivery to the eye as 0.05% oily solution (using castor or olive oil) in which CsA powder is dissolved with the help of ethanol (10% of the final formulation) or in which the contents of Neoral^®^ CsA oral soft gelatin capsules are dissolved) [4,5,6,7]. Another alternative, unaffordable to many, is Restasis^®^, a marketed nanoemulsion eye drops formulation [8]. Both oily solution and nanoemulsion have received conflicting reports on patient tolerability, ocular bioavailability, and side effects such as itching, redness, and burning sensation [4,8].

This study aimed to develop an aqueous ophthalmic, patient friendly, CsA formulation. Among preformulation issues considered was CsA poor solubility and permeability. Although hydrophobic, CsA is a class IV drug in the BCS [9], showing low solubility [10,11] and low permeability [12], due in part, to its large molecular size [11,13]. A nano-system capable of effectively encapsulating CsA, holding it in solution state and facilitating its uptake in the cornea was targeted. Several nanodelivery systems for ocular delivery of CsA providing prolonged release have been discussed [4]. Lipid nanocapsules (LNCs), relatively new biomimetic nanovectors for drug and gene delivery [14,15] were considered a good choice in the present study. They are prepared using a solvent-free method [16] and consist of an oily core surrounded by a tensioactive rigid membrane of lecithin and pegylated surfactant, providing a hybrid structure between polymeric nanocapsules and liposomes. LNCs show good colloidal properties with a size range 20–100 nm and a narrow range of dispersity [17], considered added benefit in ocular delivery [14,15,16,17,18]. Moreover, LNC dispersions are physically stable maintaining their structural integrity for long periods of time [16].

A further advantage of LNCs is their ability to encapsulate hydrophilic drugs such as fondaparinux [19] and doxorubicin [20], lipophilic drugs such as paclitaxel [21], praziquantel [22], and itraconazole [23], and amphiphilic drugs such as amiodarone [24].

LNCs were previously reported to enhance drug delivery through oral [19,23], parenteral [20], and topical [23] routes. Recently, LNCs were investigated for ocular use [25,26]. One of these studies showed that blank LNCs had no effect on viability of human corneal epithelial (HCE) cells, and did not cause corneal damage, opacity, conjunctival chemosis, conjunctival redness, vitreous haze, or retinal damage following subconjunctival injection of drug-loaded LNCs in rabbits [25].

Another preformulation issue tackled is the ease with which most aqueous ophthalmic preparations are washed out by tears. Several strategies are known to improve precorneal retention, among them in situ gels. They have the advantage of both liquids and gels; providing easy instillation as well as an improvement of precorneal retention once they attain gel state in physiological conditions [27,28].

Poloxamers are triblock copolymers widely used as non-ionic surfactants and solubilizers [29]. They are generally recognized as safe (GRAS) excipients and have been investigated for oral, rectal, ophthalmic, nasal, and vaginal use [30]. Poloxamer 407 has the lowest toxicity and low critical gelation concentration among the poloxamers and has been extensively studied for ophthalmic use [29]. Thermosensitivity of poloxamer gels is concentration-dependent; higher concentrations result in faster gelling at lower temperatures [31]. Poloxamer in situ gel exhibits pseudoplastic shear thinning flow resulting in good spreading over the cornea surface during blinking (representing periods of high shear stress), however, still being retained on the surface [32]. Poloxamer 407, widely used in ophthalmic products [29] was selected in the present study to develop in situ gels. To the best of our knowledge, LNC in situ gels were prepared for the first time in the present study.

Chitosan is considered a cationic agent of choice for ocular formulations; it is a biocompatible and biodegradable linear polysaccharide with several free amino groups imparting a positive charge capable of interacting with negatively charged cornea surface mucin. There is some evidence that chitosan might enhance cornea uptake of drug molecules [4,33]. It is thought to act as a permeation enhancer due to its mucoadhesive properties. It binds to the epithelial cells membrane and the positive charge results opening the ZO-1 tight junction [34]. Chitosan was added to the LNC in situ gel formulation to enhance corneal mucoadhesion aiming to further improve gel resistance to washing out.

The objective of the present study was to develop a thermoresponsive mucoadhesive ophthalmic gel containing CsA encapsulated in LNCs and to characterize the developed formulation in vitro to assess pharmaceutical attributes, ex vivo using excised bovine cornea to assess mucoadhesion, and in vivo in rabbits to assess biodistribution, irritation potential, and efficacy in a dry eye model.

## 2. Materials and Methods

### 2.1. Formulation Preparation

All stated concentrations refer to concentrations in the final formulations.

#### 2.1.1. Preparation of Blank and CsA-Loaded LNCs

LNCs were prepared using the phase inversion and temperature cycling method [16]. Propylene glycol dicaprylate (Labrafac^®^ lipophile WL 1349, Gattefossé S.A., Saint-Priest, France), macrogol 15 hydroxystearate (Kolliphor HS 15^®^, formerly Solutol, BASF, Ludwigshafen, Germany), deionized water (1:1:3 by weight), NaCl 0.44% *w/v* and phosphatidylcholine (Lipoid S100 0.75% *w*/*v*, Lipoïd GMBH, Ludwigshafen, Germany) were heated under magnetic stirring to 85 °C followed by cooling to 60 °C. Heating/cooling was repeated for 3 cycles at a rate of 4 °C change/min.

In the last cycle, quenching was induced by a 3.5-fold dilution using cold deionized water (2 °C) added at the phase inversion temperature (PIT) around 75 °C, detected visually when the mixture appeared translucent. The dispersion was left under slow magnetic stirring for 5 min, filtered through a 0.45 µm syringe millipore filter, and kept at 4 °C until further characterization.

For CsA-loaded LNCs (CsA, Dalian Launcher Fine Chemical Co., Ltd., Dalian, China), 10–50 mg CsA/10 mL formulation were added with LNC ingredients from the beginning.

#### 2.1.2. Preparation of Poloxamer In Situ Gels (P_in situ gels_)

Concentrations of Poloxamer 407 (P) (BASF, Germany) ranging from 17.5 to 25% *w*/*w* were prepared by dissolving in cold deionised water (4–8 °C) with continuous stirring for 3 h and refrigerated overnight [35,36].

#### 2.1.3. Preparation of Chitosan/Poloxamer In Situ Gel (CP_in situ gel_)

Chitosan (C), (molecular weight 100,000–300,000, Acros organics, part of Thermo Fisher Scientific, Geel Belgium) 0.5% *w/v* was dispersed in 1% *v/v* acetic acid solution and stirred overnight. Poloxamer (17.5 to 25% *w*/*w*) was dissolved in the chitosan solution, stirred for 3 h, and refrigerated overnight [37].

For CsA-containing in situ gels, CsA was dissolved in the cold in situ gel (P_in situ gel_ and CP_in-situ gel_), resulting in a clear solution.

#### 2.1.4. Preparation of LNC In-Situ Gels

Compatibility between poloxamer and LNC ingredients was first investigated by FTIR (Supplementary Material).

For gel preparation, poloxamer (17.5 to 25% *w*/*w*) was dispersed in cold LNC dispersion and refrigerated overnight. Quenching volume used in LNC preparation was decreased to accommodate volume increase due to added poloxamer.

For LNC-CP_in situ gel_, glacial acetic acid (1% *v*/*v*) was added to LNC dispersion. Chitosan powder (0.5% *w*/*v*) was dispersed in the acidified LNCs and stirred overnight. Poloxamer was added to the LNC-chitosan mixture.

In all in situ gel formulations prepared, final CsA concentration was 0.1% *w*/*v*. Selected formulations with CsA concentration 0.05% *w/v* were also prepared for in vivo testing.

### 2.2. In Vitro Characterization

#### 2.2.1. Characterization of In-Situ Gels

##### Determination of Gelling Temperature

Gelling temperature was measured on 2 mL samples using the inverted tube method [35]. The samples were heated from 20 °C to 70 °C at a rate of 1 °C/min.

##### Determination of Gelling Time

Time in seconds for sol-gel transition in blank in situ gels using100 µL samples was determined using an aluminum pan placed on a hot plate maintained at 37 °C. Gelation time was recorded when the sol ceased to flow when pan was tilted 90° [35].

##### Viscosity Measurement

Viscosity was measured using cone and plate viscometer (Brookfield, DV2T, USA) at 10 rpm using spindle 40 for 5 min., at 25 °C and at 37 °C. Profiles were plotted at different shear rates (1, 5, 10, 50 rpm) at 37 °C using the Rheocalc T software. Both upwards and downwards curves relating shear stress to shear rate were generated to detect possible thixotropic behavior.

#### 2.2.2. Colloidal Characterization of LNC as Dispersion and in In-Situ Gel

Mean particle size and polydispersity index (PdI) of LNCs were determined by photon correlation spectroscopy (PCS), and the zeta potential (ζ-potential) was measured by Laser Doppler Velocimetry (LDV) using a Malvern Zetasizer (Nano ZS Series DTS 1060, Malvern Instruments Ltd., Malvern, UK) at a fixed angle 173° at 25 °C using a 4 mW He-Ne laser at 633 nm. Samples were diluted 1:20 *v*/*v* with filtered deionized water prior to measurements. Data were processed using the Zetasizer software (version 7.02). The same procedure was applied for sizing LNC gels. Three samples per formulation were measured in triplicate (n = 9).

#### 2.2.3. Microscopical Examination

Images of CsA-loaded LNC dispersion and LNC-P_in situ_ *_gel_* at optimized poloxamer concentration were taken by transmission electron microscopy (TEM, Jeol, JEM-100 CX Electron Microscope, Tokyo, Japan) preceded by negative staining for 30 s of 4-fold diluted samples with deionized water using 2% *w*/*v* aqueous uranyl acetate solution. Photographs were taken at 30–40 K X magnification power at 80 kV.

### 2.3. Entrapment Efficiency (%EE) and Drug Loading

Percent EE was determined by measuring free (un-entrapped) CsA separated from LNC dispersion using an ultrafiltration/centrifugation technique for 30 min at 3663× *g* at 25 °C (SartoriusTM Vivaspin 6, MWCO 100,000) [38]. For in situ gels (LNC-P_in situ gel_ and LNC-CP_in situ gel_), 1:4 dilution with deionized water was done prior to ultracentrifugation. Entrapped CsA was calculated from the difference between total CsA concentration (Ct) and free concentration (Cf), divided by Ct.

To determine Ct, LNC dispersion (100 μL) was diluted with methanol (up to 2 mL), sonicated for 20 s and CsA assayed by a validated HPLC method (Supplementary Material).

Drug payload was calculated by dividing Ct by dry weights of the LNC ingredients. The effect of initial amount of CsA on EE and on LNC properties was also determined.

### 2.4. Stability Testing of LNC Dispersion and LNC In-Situ Gels

Formulations were refrigerated for 6 months at 4 °C. Particle size, PdI, and ζ-potential were assessed monthly during the first three months and after 6 months by Malvern Zeta sizer. Changes in%EE were also monitored.

### 2.5. Ex Vivo Mucoadhesion Study

Mucoadhesion force was measured using the modified physical balance method [39]. Bovine eyeballs were obtained from a local slaughterhouse and used on the same day of ablation. Cornea preparation followed the reported procedure [40]. Method details are provided in Supplementary Material.

### 2.6. In Vivo Studies in Rabbits

#### 2.6.1. Ethics Statement

Animal studies were conducted at the Animal Experimentation Facility, Faculty of Pharmacy Alexandria University, Egypt, in accordance with Principles of Laboratory Animal Care and institutional Guidelines for ethical conduct in use of animals in research. Efforts were made to minimize animal suffering. The study protocols were approved by the institutional Ethics Committee.

#### 2.6.2. Animals

Male New Zealand colored rabbits weighing 1.2–1.5 kg and free from any visible ocular damage were selected. Rabbits were provided with food and water in a temperature-controlled room (25 °C).

#### 2.6.3. Corneal Biodistribution Study

DiI (1,1-Dioctadecyl-3,3,3,3 tetramethyl indocarbocyanine perchlorate, Sigma-Aldrich, USA) was used for fluorescent labeling. Labeling method is provided in Supplementary Material.

A total of 4 rabbits were used. Fifty microliters of fluorescent -labeled blank formulations were instilled in lower conjunctival sac (DiI-LNC dispersion, DiI-LNC-CP_in situ gel_, DiI-castor oil and DiI solution as control). Rabbits were sacrificed 3 h post administration and the eyes were enucleated and stored in 10% *v/v* formalin. Histological sections, 5 µm thick, were prepared for laser scanning confocal microscopy (detailed in Supplementary Material).

#### 2.6.4. Efficacy in the Dry Eye Rabbit Model

Schirmer Tear test (STT) [41] was used. Eighteen rabbits were randomly divided into 6 equal groups. CsA-LNC-CP_in situ gel_ (0.05% and 0.1% *w*/*v*) was compared with marketed CsA-NE and with CsA solution in castor oil (0.05%). Positive (untreated) and negative (receiving normal saline) control groups were included.

Using Schirmer tear test strips, tear production was monitored over a total of 10 days. Induction of dry eye (starting on day 0) was done using 50 μL of 1% atropine sulphate (AS) eye drops, three times daily instillation in both eyes for 3 days [42]. This was followed by daily treatment using the CsA formulations (50 μL) from day 4 to 10, half an hour after instillation of 1% AS. Tear production was assessed on day 0 (baseline) and on day 3 following AS administration for 3 days, as well as on days 5, 7, and 10. The STT strip (Eye Care Products Delhi, India) was placed inside the margin of the lower eyelid for two min and the level of strip wetting was noted. The height of the strip wetted by the tears was recorded in millimeters.

#### 2.6.5. Ocular Irritation Assessment in Rabbits

Nine rabbits were divided into three equal groups given either 50 µL of CsA-LNCs-CP_in situ gel_ (0.1%) or CsA-NE or CsA eye drops in castor oil. Self-control method was adopted; right eye was treated, and left eye was given the same volume of normal saline solution. Rabbits were treated twice daily for 14 days. Visual inspection of the eye took place over 14 days for any signs of ocular reaction (redness, discharge, swelling, iris hyperemia, and corneal lesions or opacity) and continued for three days after stopping treatment [43]. Draize scoring system was applied.

Rabbits were euthanized with an intravenous air injection and eyeballs were enucleated for histopathological examination using a light microscope. Enucleated eyeballs were fixed in 10% formalin. Five-micrometer-thick histological sections were stained with hematoxylin and eosin.

### 2.7. Statistics

Data comparison was performed by one-way ANOVA followed by pairwise comparisons (Tukey test) using SPSS 20.0; (SPSS Inc., Chicago, IL, USA). Significant differences were considered at *p* ≤ 0.05.

## 3. Results and Discussion

LNCs were successfully prepared using the phase inversion method [16] with no modification and resulted in LNC with good quality attributes.

### 3.1. LNC Characteristics

**Figure 1:**
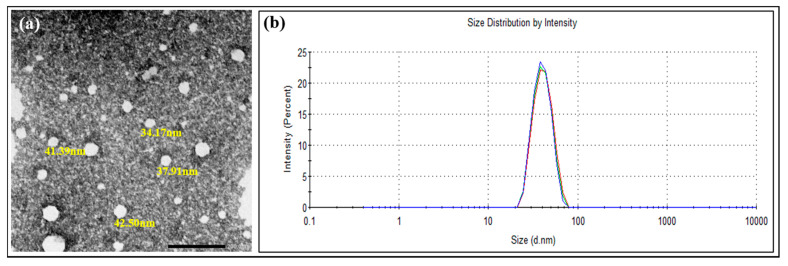
(**a**) TEM image of CsA-loaded lipid nanocapsules (LNCs) × 30,000. The scale bar represents 200 nm. (**b**) Size distribution by intensity curve of CsA-loaded LNC dispersion.

Mean particle size of blank LNCs was 39.75 ± 1.48 nm, PdI 0.04, and zeta potential −3.2 ± 2.1 mV; the negative charge is most likely due to the presence of small proportion of hydrolyzed surfactants [44]. Loading 10 mg CsA (0.1% *w*/*v*) resulted in minimum change in colloidal properties (size 41.9 ± 4.0 nm) and zeta potential −4.0 ± 1.50 mV. PdI value remained less than 0.1 for 10–50 mg initial CsA. CsA-loaded LNCs were spherical in shape and monodispersed (Figure 1a,b).

### 3.2. HPLC Data

CsA was assayed by HPLC; peak areas were linear over concentration range 0.5–25 µg/mL, r^2^ ≥ 0.99 (Supplementary Material, Figure S1). Heating the column to 80 °C sharpened the peak [45]. Limits for detection and quantification were 0.132 µg/mL and 0.438 µg/mL, respectively. Inter- and intra-day precision for standard CsA solutions were 9.45 ± 3.54% and 1.24 ± 0.16%, respectively, as an indication of precision, with inter- and intra-day mean percent recovery of 99.13% and 98.97%, respectively, as an indication of accuracy.

### 3.3. CsA Loading and Entrapment Efficiency (EE)

At initial CsA amount (10 to 50 mg/10 mL dispersion), determined total CsA (free and entrapped) ranged from 9.6 ± 0.6 to 47.6 ± 4.2 mg. EE was constant and above 98% (calculated from determined total and free CsA), and CsA payload ranged from 0.83% to 4.18%. Beyond 50 mg initial CsA, signs of drug precipitation were evident.

High entrapment of CsA is mostly due to its incorporation in the oily core of LNCs due to its lipophilic nature (log P = 3). Similar results have been previously reported with lipophilic drugs in LNCs, such as paclitaxel [21], ropivacaine [46], and praziquantel [22]. CsA concentration targeted in the final formulation was 0.1% (10 mg/10 mL). Higher CsA concentrations (up to 50 mg/mL) successfully loaded in LNCs could prove useful in veterinary ophthalmic CsA products requiring higher CsA concentrations.

### 3.4. Gel Characteristics (P_In Situ gel_ and LNC-P_In Situ gel_)

Gelling data (Table 1) identified optimum P concentration for ocular P_in situ gel_ and assessed effects of chitosan and LNCs on gelling temperature, gelling time, and viscosity. The optimum in situ gels identified (shaded in Table 1) were selected for further ex vivo and in vivo assessment.

Increasing P concentration resulted in a decrease in gelling temperatures (*p* < 0.005). Similarly, addition of Chitosan (0.5%), resulted in a significant decrease in gelling temperature (at P 20 to 25%) (Table 1). P (25% *w*/*w*) formed gel at 34.8 ± 0.32 °C, consistent with reported data stating that 25% w/w Pluronics are required to form a stiff gel upon ocular administration [47,48].

LNC- P_in situ gel_ showed lower gelling temperature than P_in situ gel_ at all P concentrations (Table 1); increased P solution viscosity by LNCs nano-state probably contributed. Similarly, solid lipid nanoparticles in P gel, demonstrated decreased gelling temperature and gelling time as well as increased gel viscosity and mechanical strength [49]. A similar observation was reported by Nie et al. [36], where the addition of liposomes to P resulted in a decrease in gelling temperature.

**Figure 2:**
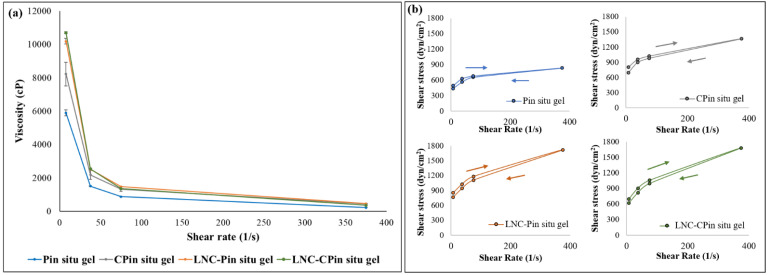
(**a**) Rheogram of blank in situ gels at physiologic temperature. (**b**) Thixotropic behavior of in situ gels.

Rheograms constructed showed shear thinning behavior of the in situ gels (Figure 2a), which is considered optimal for ocular administration; ocular shear rate ranges from 0.03 s^−1^ during inter-blinking periods to 4500–28500 s^−1^ during blinking. The shear thinning property will allow good distribution of the formulation over the ocular surface during blinking. In addition, during inter-blinking periods, high viscosity will maintain prolonged contact between gel and corneal surface [31,32,50]. In situ gels showed thixotropic behavior (Figure 2b), with the downwards curves exhibiting lower shear stress compared to corresponding points on the upwards curves. The inclusion of LNCs did not apparently affect the observed hysteresis loops.

### 3.5. Effect of Poloxamer on LNC Colloidal Properties

**Figure 3:**
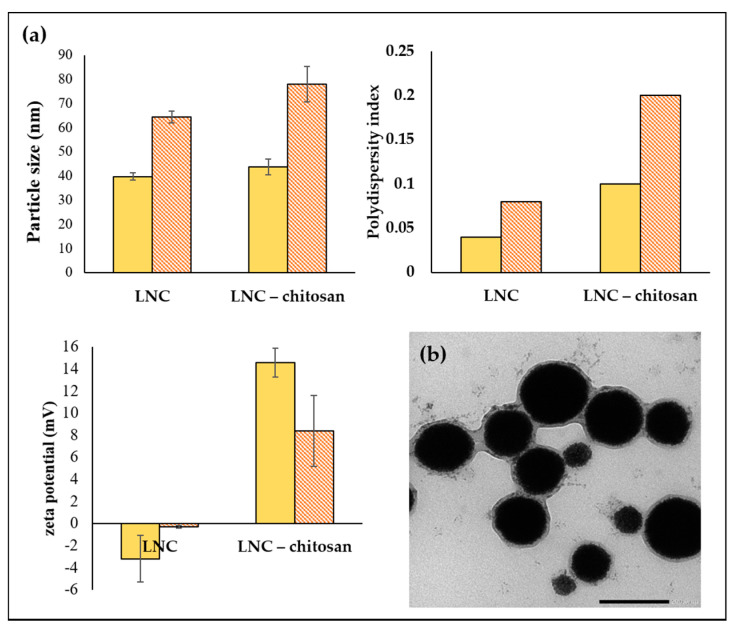
Effect of poloxamer on (**a**) blank LNC and blank LNC-chitosan colloidal properties and (**b**) on morphology of blank LNC. A poloxamer sheath surrounding the LNCs can be seen in Figure 3b at 40,000 magnification.

Size enlargement was evident for both LNC and LNC-Chitosan after addition of P (Figure 3a). TEM image of LNC-P confirmed increase in size (Figure 3b).

When comparing each in situ gel with its respective dispersion there is a significantly higher PdI (*p* < 0.01) (Figure 3a), which remained, however, less than 0.2 indicating mono-dispersity of LNC-P_in situ gel_ and LNC-CP_in situ gel_. This increase in PdI could be attributed to the adsorption of P on the LNCs’ surface, not to particle agglomerates as previously suggested by Hao et al. for SLNs [51]. Addition of poloxamer to LNC formulations (both LNC and LNC-C) showed a significant decrease in surface charge when comparing the in situ gel with its respective dispersion (*p* < 0.05) (Figure 3a). This could also be due to the formation of the poloxamer sheath surrounding the LNCs, masking the surface charge. Nanostructured lipid carriers in 20% P also showed a marked decrease in zeta potential [52].

### 3.6. Compatibility of Poloxamer with LNC Ingredients

No incompatibility was detected by FTIR between P and LNC ingredients (Supplementary material, Figures S2–S4). Poloxamer has been previously used as an in situ gelling agent incorporating nanoparticles. Nie et al. [36] prepared liposomes containing paclitaxel dispersed in18% P thermoreversible gel. Ketoconazole loaded nanoparticles were also dispersed in 16% [50]. Desai et al. [53] prepared polyisobutylcyanoacrylate nanocapsules dispersed in 25% P with 5% methylcellulose.

### 3.7. Storage Stability Data at 4 °C of CsA-LNC_dispersion_ and LNC_in situ gel_

**Figure 4:**
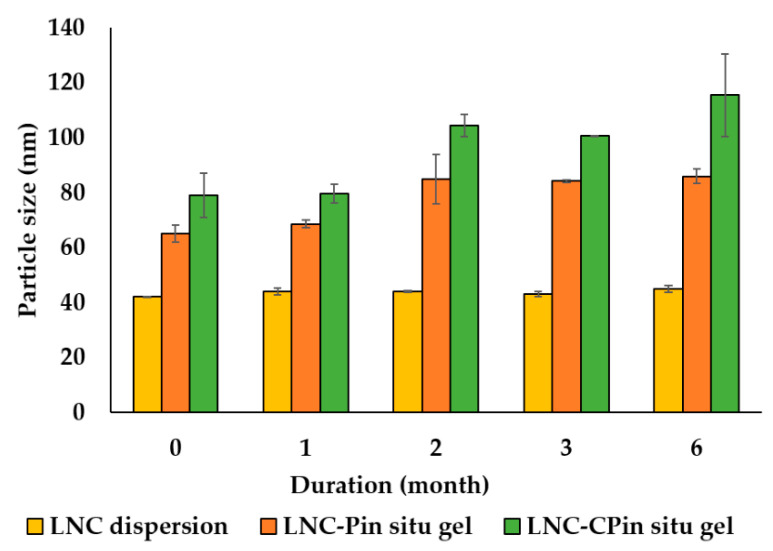
Particle size (nm) of CsA loaded LNC dispersion and in situ gels during storage at 4 °C over time. (n = 3).

Particle size, PdI, ζ—potential, and entrapment efficiency were monitored over six months (Figure 4). Particle size of freshly prepared CsA-LNC _dispersion_ showed marginal increase from 42.2 ± 0.2 to 44.9 ± 1.4; corresponding data for LNC- P _in situ gel_ and LNC-CP _in situ gel_ were 64.96 ± 3.1, and increased to 85.9 ± 2.6 and 79.1 ± 8.1 to 115.45 ± 15.0, respectively (*p* < 0.05, in case of in situ gels). Changes were evident in the first two months and tended to stabilize beyond. A possible explanation for apparent size enlargement in case of in situ gels is progressive hydration of poloxamer sheath surrounding LNCs, while LNC size remained relatively unaffected, as seen with LNC _dispersion_.

PdI followed the same pattern as the particle size; in situ gels remained monodispersed over the storage period with pdI < 0.25 (*p* > 0.05). ζ-potential data for LNC-CP_in situ gel_ indicated no significant change over six months (6.5 ± 1.0 and 7.1 ± 0.6 mV at 0 and six months, respectively). Similarly, % EE showed no significant change over six months (*p* > 0.05); all formulations remained above 98% EE.

ζ-potential values beyond ± 30 mV are considered required for electrostatic stabilization; systems with values between ± 5 to ±15 mV are in the limited flocculation region while those between ±3 to ±5 mV are likely to flocculate [54]. This rule may not apply to nano-systems such as LNCs with steric stabilizers, where lower ζ—potential can provide sufficient stabilization [54]. LNCs with low negative ζ-potential stored at 4 °C were reported stable for over 18 months and those stored at 37 °C were stable for 1.5 months [16].

### 3.8. Ex Vivo Results

#### Mucoadhesive Force

Data assessing mucoadhesive force at 37 °C, of CsA-loaded LNCs _dispersions_ and LNC _in-situ gels_ expressed as the force required to detach the formulation from the corneal surface [31], were generated using the modified physical balance method [39], the formulation was applied between two face-to-face placed bovine corneas.

LNC _dispersion_ showed lowest mucoadhesive force (338.1 ± 36.5 dyne/cm^2^). Chitosan (0.5% *w*/*v*) addition to LNC _dispersion_ increased mucoadhesive force to 629.4 ± 146.9 dyne/cm^2^, (*p* > 0.05). Value recorded for LNC-P _in situ gel_ was 2127.5 ± 377.3 dyne/cm^2^ (*p* < 0.001, vs. LNC _dispersion_), due to increased gel viscosity at the study temperature, and also to the ability of polymers with hydrophilic functional groups such as hydroxyl groups in P to form electrostatic and hydrophobic interactions as well as hydrogen bonding with cornea surface [55,56].

Addition of chitosan to the in situ gels further increased mucoadhesive force (LNC-CP_in situ gel_, 3412.3 ± 442 dyne/cm^2^, *p* <0.001, vs. LNC-P _in situ gel,_) due probably to ionic interaction between chitosan amino group and anionic substructures of cornea mucus layer [57]. The increase in mucoadhesive force observed suggests that both P and C are present on the surface of the LNCs.

### 3.9. In Vivo Data

#### 3.9.1. Corneal Biodistribution

Confocal microscopy using a fluorescent dye offers an attractive alternative to analyzing drug in cornea layers when faced with the limitation of the analytical method. In the case of CsA, the sensitivity of the HPLC method did not allow assessing CsA levels in the successive cornea layers at 3 h post instillation in the rabbits.

The choice of Dil as a lipophilic fluorescent dye to act as CsA surrogate was based on results of several published studies providing proof of fluorescent dye labeling stability in lipid nanocapsules when using lipophilic indocarbocyanines (DiO, DiI, and DiD) incorporated in LNCs compared to other fluorescent substances [58,59].

**Figure 5:**
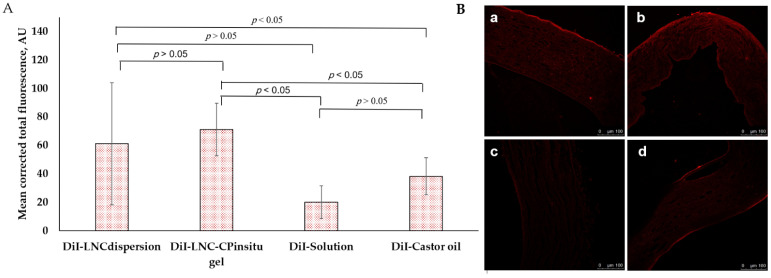
In vivo corneal biodistriution 3 h post administration showing: (**A**) Mean corrected total fluorescence of corneas following administration of different DiI formulations and (**B**) confocal images of cornea layers following administration of (**a**) DiI-LNC _dispersion_, (**b**) DiI-LNC-CP_in situ gel_, (**c**) control DiI-Solution, and (**d**) DiI-Castor oil, scale bar 100 µm.

Cornea distribution of fluorescent DiI was observed by confocal laser microscopy (Figure 5). Based on fluorescence intensity (Figure 5A), highest accumulation was achieved by DiI-LNC-CP_in situ gel_ followed by DiI-LNC_dispersion_ (*p* > 0.05). Values for DiI-Castor oil and control DiI solution were lower. The reported stability of DiI in LNCs [58,59] suggested that the data reflected the biodistribution of LNC entrapping Dil.

DiI-LNC formulations showed strong fluorescence in cornea layers, with fluorescence in corneal epithelium higher than in stroma (Figure 5B(a,b)). Control DiI solution confocal images (Figure 5B(c)) showed limited fluorescence in corneal layers due, possibly, to the hydrophobic nature of the corneal epithelium. DiI-Castor oil confocal images showed fluorescence in corneal epithelium with limited to no fluorescence in stroma (Figure 5B(d)). Results suggested the presence of LNCs enhanced DiI dye penetration and accumulation in cornea, possibly serving as drug reservoir.

The positive effect of drug encapsulation in LNCs on corneal accumulation could be partly justified by their small particle size (<50 nm); previous reports have shown that nanocarrier particle size is inversely proportional to membrane permeability with particles below 400 nm showing greater penetration through polarized epithelial cells [60,61]. The amphiphilic nature of LNCs could probably enable them to permeate through the hydrophobic corneal epithelium and endothelium and to accumulate within the hydrophilic stromal layer [62]. Additionally, the polyoxyethylene-type nonionic surfactant surface displays a P-gp efflux pump inhibitory effect expressed on the surface of corneal epithelial cells, believed to be a major barrier to drug delivery [63].

Corneal accumulation may also be due to infiltration mechanism, where the nanocarrier (polycaprolactone nanospheres) were claimed to be absorbed onto the corneal surface, followed by transport of encapsulated drug into the corneal epithelial cell membrane [64].

#### 3.9.2. Comparative In Vivo Efficacy in the Dry Eye Rabbit Model

Schirmer Tear Test (STT) without anesthesia (type I) was used to measure aqueous tear secretion in response to both conjunctival stimulation and basal non-reflex secretion. Dry eye induction model was selected; it is simple and able to rapidly induce dry eye (DE) symptoms compared to other invasive models. Burgalassi et al. [65] reported that decreased tear production can be expected on the second day of topical atropine sulphate (AS) instillation evident by decreased STT values, due to antimuscarinic effect on lacrimal gland’s innervations by parasympathetic fibers of the seventh cranial nerve.

Baseline tear production was recorded on day 0 before AS instillation; average tear production was 10.48 ± 4.3 mm, n = 36 (18 rabbits, both eyes). Differences between the study groups were insignificant (*p* < 0.4). Tear production was then followed over a period of 10 days (Table 2).

The negative control group (3 rabbits) receiving normal saline drops maintained a consistent STT value over 10 days (11 mm ± 2.65). A significant reduction in tear production (*p* < 0.01, compared to day 0 values) was recorded for the other five animal groups (15 rabbits) receiving 1% AS eye drops three times daily, measured on day 3 indicating successful induction of DE, maintained over the study period by three times daily instillation of 1% AS (maintenance evident in untreated positive control group data, Table 2).

Starting from day 5, an increase in STT value was observed for the four treated groups. Compared to day 3 values, changes were statistically insignificant (*p* > 0.5) for both CsA-NE and CsA/castor oil treated groups (values changed from 2.0 ± 1.7 mm and 4.3 ± 2.3 mm on day 3 to 6.0 ± 3 mm and 5.0 ± 1.7 mm on day 5, respectively). LNC-CP_in situ gels_ treated groups showed a statistically significant (*p* < 0.005) increase in STT values which changed from 5.5 ± 2.5 and 3.8 ± 0.8 mm on day 3 to 10.5 ± 4.2 and 12.4 ± 4.3 mm on day 5 for 0.05% and 0.1% CsA, respectively (Table 2).

**Figure 6:**
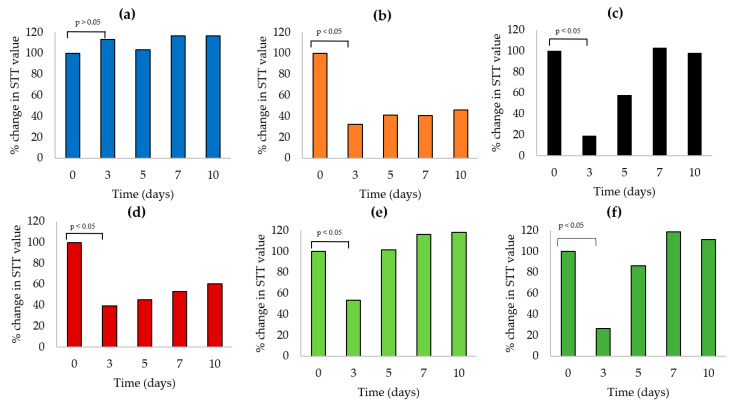
Change in Schirmer tear test values (day 0 values expressed as 100%), in rabbits over a 10-day period after daily instillation of (**a**) 0.9% saline only, or 1% AS and either (**b**) not treated or treated with (**c**) CsA-NE, (**d**) CsA in castor oil, (**e**) CsA-LNC-CP_in situ gel_ (0.05% CsA), or (**f**) CsA-LNC-CP_in situ gel_ (0.1% CsA).

Figure 6 (data expressed as percent tear production, with reference to day 0 values) allowed judging comparative efficacy. Judging by recovery of baseline tear value, three of the four tested formulations (two LNC formulations and CsA-NE) produced 100% recovery by day 7; CsA in castor oil failed to bring about complete recovery of base line tear value by day 10.

Concerning comparative rate of recovery (difference in percent tear production from day 3 to day 5, Figure 6), the values were 60%, 48%, and 39% for 0.1% CsA-LNC-CP_in situ gel,_ 0.05% CsA-LNC-CP_in situ gel_, and CsA-NE, respectively. With CsA in castor oil, the rate of improvement was less than 5%.

In vivo efficacy of 0.05% and 0.1% CsA-LNC-CP_in situ gel_ could be partly attributed to gel mucoadhesive properties enhanced by interaction between positively charged chitosan and negatively charged cornea mucin layer, hence increased CsA-LNC retention on cornea surface.

CsA in castor oil failed to demonstrate comparative therapeutic response in the dry eye rabbit model; it is not clear why baseline tear values were not reached within seven days of initiating treatment. Coating of conjunctiva with residual oil could have prevented proper functioning of the Schirmer tear strip although measurements were taken one hour after instillation of all treatment formulations to allow for partial formulation clearance from conjunctiva. Alternatively, poor tolerance by the eye, rapid clearance from ocular surface [64,66] in addition to poor corneal uptake (demonstrated in the present ex vivo study) could be responsible for the observed lower efficacy of castor oil formulation.

The Schirmer tear test was able to demonstrate effectiveness of the LNC formulations under the same conditions as the marketed CsA-NE but could not demonstrate greater effectiveness of the 0.1% compared to the 0.05% LNC formulation; both gave similar results. The 0.1% concentration is recommended for severe cases of DE, which may not apply to the dry eye rabbit model.

#### 3.9.3. Ocular Irritation Test

Modified Draize test [67] was conducted to assess irritation potential of 0.1% CsA-LNC-CP _in situ gel_ in comparison with marketed CsA-NE and CsA/castor oil eye drops.

No behavioral reaction such as eye rubbing was observed in all animals tested following twice daily instillation of any of the three formulations studied. Rabbits showed no symptoms of ocular irritation such as redness, tearing, inflammation, or swelling following twice daily instillation over a period of two weeks. The eye lids showed healthy rosy color with no swelling, and the cornea and conjunctiva showed no pathological alterations in comparison to the control eye receiving only NSS.

**Figure 7:**
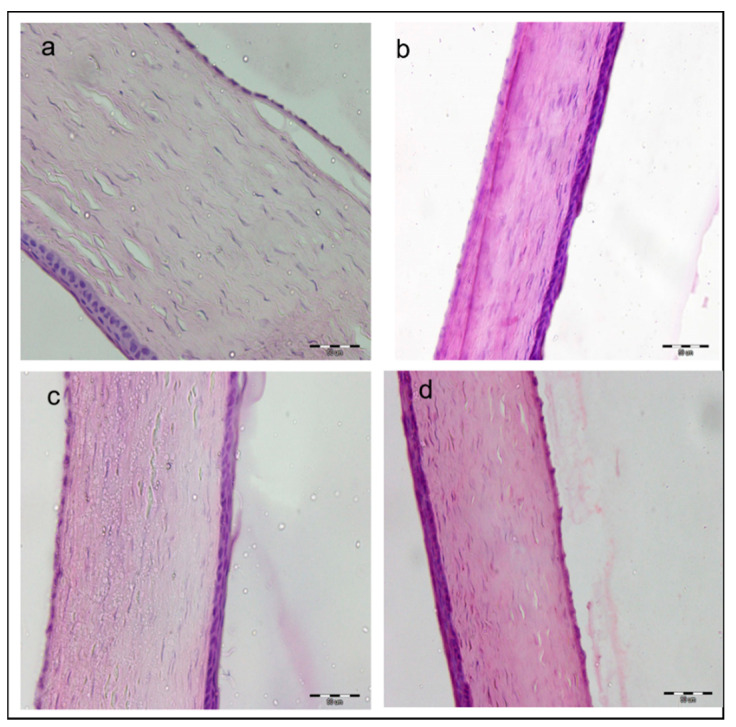
Histopathologic evaluations of rabbit corneas after 14 days of twice daily administration of (**a**) normal saline, (**b**) CsA-NE, (**c**) CsA in castor oil and (**d**) 0.1% CsA-LNC-CP _in situ gel_. scale bar 50 µm.

To further validate observational results, animals were sacrificed, eyeballs were removed, and histopathological examination of the ocular tissues was done after H & E staining. No signs of changes at cell and tissue levels were detected (Figure 7). Photomicrographs showed no vessel proliferation or immune cell infiltration. The cornea also had an unchanged appearance in all its layers with no angiogenesis or inflammatory signs when compared with published normal bovine cornea photomicrographs [68].

## 4. Conclusions

A mucoadhesive thermoresponsive ophthalmic in situ gel containing CsA loaded in LNCs was developed and characterized. In vitro, ex vivo, and in vivo data generated provided evidence that CsA- LNC-CP_in situ gel_ could serve as an effective patient-friendly formulation for the management of dry eye condition. Results of the present study provide incentive for a future clinical study to determine efficacy and tolerability in humans. The inclusion of chitosan in the gel enhanced mucoadhesion and offers a positively charged promising LNC formulation, possibly useful in other applications.

## Figures and Tables

**Table 1:** Gelling properties and viscosity of blank P_in situ gels_ and LNC-P_in situ gels._

In Situ Gel	Poloxamer% *w*/*w*	Chitosan% *w*/*v*	Gelling Temperature, °C	Gelling Time, (Seconds)	Viscosity at 25 °C (Centipoise)	Viscosity at 37 °C (Centipoise)
**P_in situ gel_**	17.5	-	63.8 ± 0.59	>120	1.74 ± 1.5	3.49 ± 4
	20	-	46.4± 1.65	>120	6.97 ± 1.5	16.57 ± 4
	22.5	-	39.9 ± 0.75	71.8 s ± 7.5	13.08 ± 2.62	174.4 ± 41
	25	-	34.8 ± 0.32	15.77 s ± 1.28	31.39 ± 5.23	1143 ± 199
**CP_in situ gel_**	17.5	0.5	^a^	>120	33.13 ± 4	32.3 ± 4
	20	0.5	42.6 ± 0.50	>120	42.7 ± 4	43.6 ± 14.4
	22.5	0.5	36.9 ± 0.29	51.74 s ± 1.6	66.27 ± 4	235.5 ± 50.9
	25	0.5	31.8 ± 0.91	12.65 s ± 0.55	101.2 ± 8.4	1252.2 ± 163
**LNC-P_in situ gel_**	17.5	-	39.5 ± 0.2	57.6 s ± 6.37	34.88 ± 4	240.7 ± 66.7
	20	-	34.0 ± 0.4	11.5 s ± 0.6	94.17 ± 7.4	1087.1 ± 254.9
	22.5	-	32.6 ± 0.1	8.9 s ± 0.93	266.8 ± 4.5	1787.7 ± 200
	25	-	30.0 ± 1.35	^b^	1021.8 ± 216.7	^c^
**LNC-CP_in situ gel_**	17.5	0.5	45.1 ± 0.56	46.5 s ± 4.3	66.27 ± 3	375.4 ± 25.1
	20	0.5	34.2 ± 0.47	11.4 s ± 0.26	136.9 ± 15.9	1315.5 ± 129.4
	22.5	0.5	28.9 ± 1.15	6.1 s ± 0.75	342.7 ± 32.77	2002.3 ± 96.7
	25	0.5	24.6 ± 1.0	^b^	1598.5 ± 103.9	^c^

^a^ Beyond 70 °C; ^b^ Gel at room temperature; ^c^ Out of range of viscosities.

**Table 2:** Schirmer tear test values of rabbits receiving 0.9% saline only for 10 days (negative control), or 1% AS for 10 days and either not treated (positive control) or treated starting on day 4 with CsA-NE (0.05%), CsA in castor oil (0.05%), or CsA-LNC-CP_in situ gel_ (0.05 and 0.1%).

Group Days		STT Value ± SD (mm)				
	Saline	AS	AS/ CsA-NE	AS/CsA in Castor Oil	AS/0.05% CsA-LNC-CP_in situ gel_	AS/0.1% CsA-LNC-CP_in situ gel_
0	10 ± 1.0	6.8 ± 1.3	10.3 ± 1.5	11.0 ± 4.0	10.3 ± 2.6	14.4 ± 6.1
3	11.3 ± 3.1	2.2 ± 0.4	2.0 ± 1.7	4.3 ± 2.3	5.5 ± 2.5	3.8 ± 0.8
5	10.3 ± 3.8	2.8 ± 1.1	6.0 ± 3.0	5.0 ± 1.7	10.5 ± 4.2	12.4 ± 4.3
7	11.7 ± 3.2	2.8 ± 1.2	10.7 ± 3.3	5.8 ± 0.8	12.0 ± 2.45	17.1 ± 4.7
10	11.7 ± 3.2	3.1 ± 0.9	10.2 ± 3.9	6.7 ± 3.0	12.2 ± 2.0	16.0 ± 3.7

## Supplementary Materials

The following are available online at https://www.mdpi.com/1999-4923/13/3/360/s1, Figure S1. Chromatogram and Calibration curve of CsA by HPLC-UV assay at 210 nm. Figure S2. IR spectra of (a) kolliphor, (b) labrafac and (c) poloxamer 407. Figure S3. IR spectra of physical mixture of (a) kolliphor-poloxamer 407, (b) labrafac-poloxamer 407 and (c) kolliphor-labrafac-poloxamer 407. Figure S4. IR septra of (a) blank LNC and (b) blank LNC in P407 in situ gel.
