# Supplementary material for: Cyclosporine Lipid Nanocapsules as Thermoresponsive Gel for Dry Eye Management: Promising Corneal Mucoadhesion, Biodistribution and Preclinical Efficacy in Rabbits
Source: Pharmaceutics. 2021 Mar 9;13(3):360. doi: 10.3390/pharmaceutics13030360 (PMC8001470; doi:10.3390/pharmaceutics13030360)
Supplement: Supplementary file 1 [file pharmaceutics-13-00360-s001.pdf]

pharmaceutics

Supplementary Materials: Cyclosporine Lipid Nanocapsules as Thermoresponsive Gel for Dry Eye Management: Promising Corneal Mucoadhesion, Biodistribution and Preclinical Efficacy in Rabbits

Lubna M. Eldesouky, Riham M. El-Moselmany *, Alyaa A. Ramadan, Mahmoud H. Morsi and Nawal M. Khalafallah

Supplementary Methods

Fourier-transform infrared spectroscopy (FTIR)

To ensure compatibility of lipid nanocapsules (LNCs) components with poloxamer 407 (P), possible interaction was investigated by FTIR. Spectra were recorded on Agilent technologies Carry 630 FTIR in the range between 4000 and 500 cm^{-1}. The instrument allows analysis of both solid and liquid samples. Spectra of individual LNC components, P, and physical mixtures of them, as well as LNC dispersions and LNC-$P_{in\ situ\ gel}$ were obtained.

HPLC assay of CsA

A reverse C18 column (4.6×250 mm, 5 µm, ZORBAX SB-C18, Agilent, USA) was used. The mobile phase was a mixture of acetonitrile: water (80:20, v/v), eluted at a flow rate of 1 mL /min. The HPLC system was equipped with an LC VL pump, UV-variable wavelength detector (set at 210 nm), and Agilent ChemStation® software 32-bit version (revision B.04.03). The column temperature was controlled at 80°C using an HPLC column temperature controller (Thermasphere® TS-130; Phenomenex, Torrance, CA, USA). Drug retention time was 6 minutes. The method was validated for accuracy and precision.

Ex vivo mucoadhesion study

Two corneas were used; a section of freshly excised bovine cornea (3.14 cm^2) was stuck (using a drop of super glue) to a metal rod connected to the balance while the second cornea was stuck on the outside of an inverted beaker placed in a wider beaker containing water not reaching the cornea, acting as a water bath. The temperature was maintained using a hot plate set at 37℃. A 100 µl formulation sample (dispersion or *in situ* gels) was added onto the cornea attached to the inverted beaker and the height of the second cornea was adjusted so that the mucosal surfaces of both corneas came into contact. The mucosal surfaces were held in contact for 2 min before weights were put onto the pan until the corneas detached. The minimum weight required to detach the two corneas was recorded and the mucoadhesive force expressed as the detachment stress in dynes/cm^2 was calculated by the following equation:

$$\text{Mucoadhesive strength} = \frac{\text{m. g}}{A}$$

where, m is the weight required for detachment (grams), A is the area of cornea mucosa exposed (cm^2), g is the acceleration due to gravity (980 cm/ s^2)

Preparation of fluorescent- labeled formulations

A stock solution (1 mg/mL) of DiI (1,1-Dioctadecyl-3,3,3,3 tetramethyl indocarbocyanine perchlorate, Sigma-Aldrich, USA) in acetone was prepared. 0.1 mL of stock was transferred to a glass vial, protected from light and heated gently to evaporate acetone. Blank LNC ingredients, were added to prepare $LNC_{dispersion}$ and LNC-$CP_{in\ situ\ gel}$ using phase inversion method. For DiI in castor oil, 10 mL castor oil were added to the DiI residue and stirred for an hour. The control was prepared by dissolving DiI in a mixture of acetone and water in a ratio of 0.1:9.9, respectively.

The final dye concentration in all formulations and control solution was 0.01 mg/mL.

Confocal Microscopy

Laser scanning confocal microscopy (LSCM) was used to identify location of DiI in the examined tissues. The microscope is equipped with a 63.0 ×HCX PL APO oil immersion objective. Four different sections of each layer per eye

were examined, and a minimum of three fields were imaged and recorded per section. Images were taken using identical parameters for each layer. The intracellular fluorescence of DiI was observed with excitation at 545 nm and emission at 550-600 nm. Confocal images were analyzed using Image J software (version 1.45s). Mean corrected total fluorescence (MCTF) for identical areas of the analyzed image was calculated by subtracting background autofluorescence.

Supplementary Figures

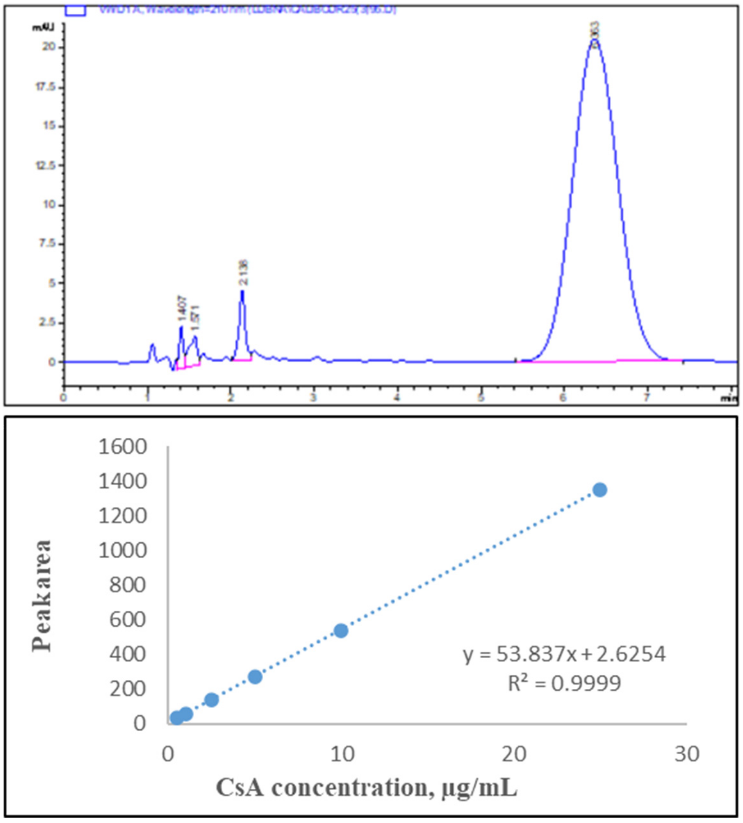

Figure S1. Chromatogram and Calibration curve of CsA by HPLC-UV assay at 210 nm.

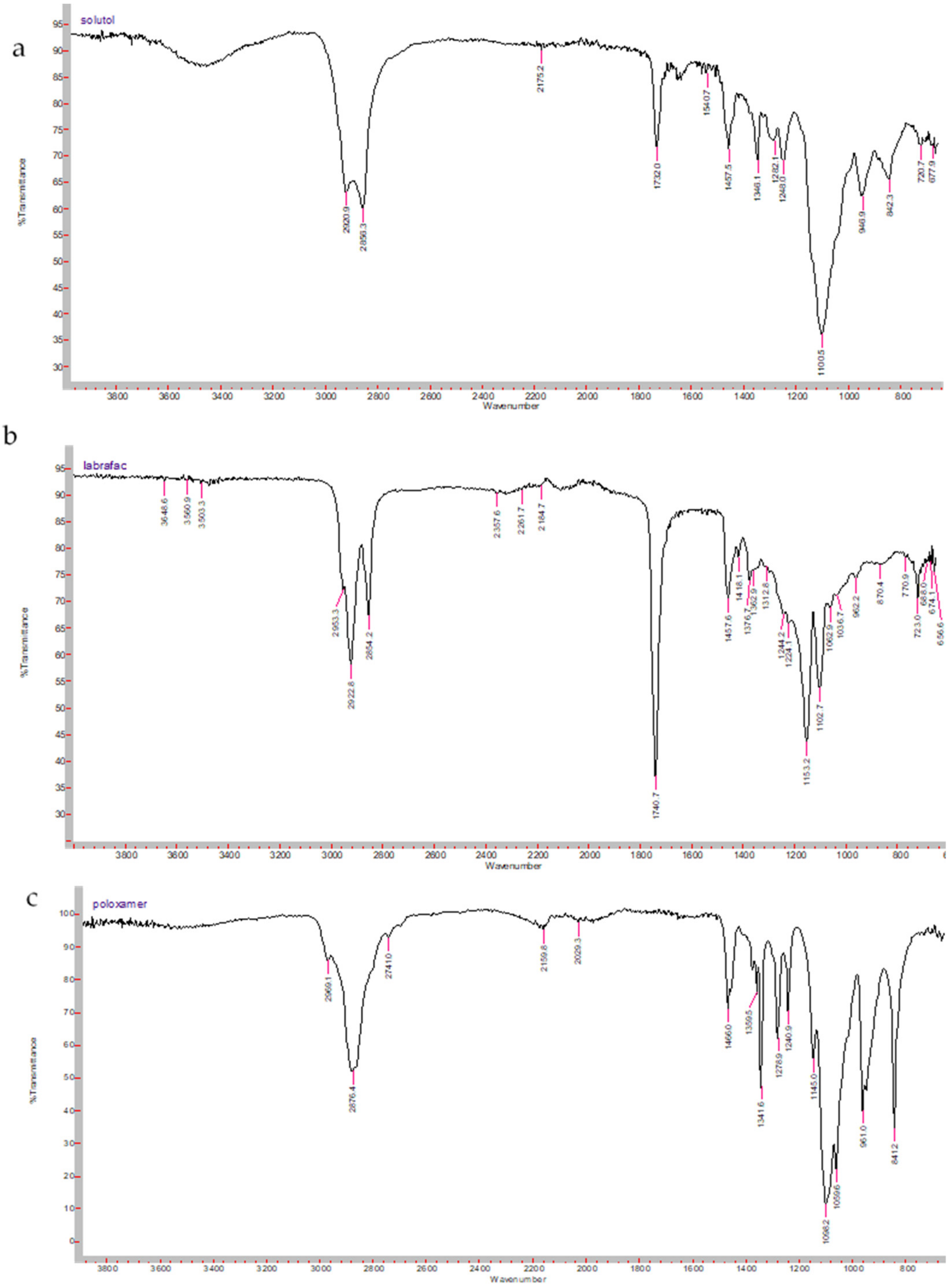

Figure S2. IR spectra of (a) kolliphor, (b) labrafac and (c) poloxamer 407.

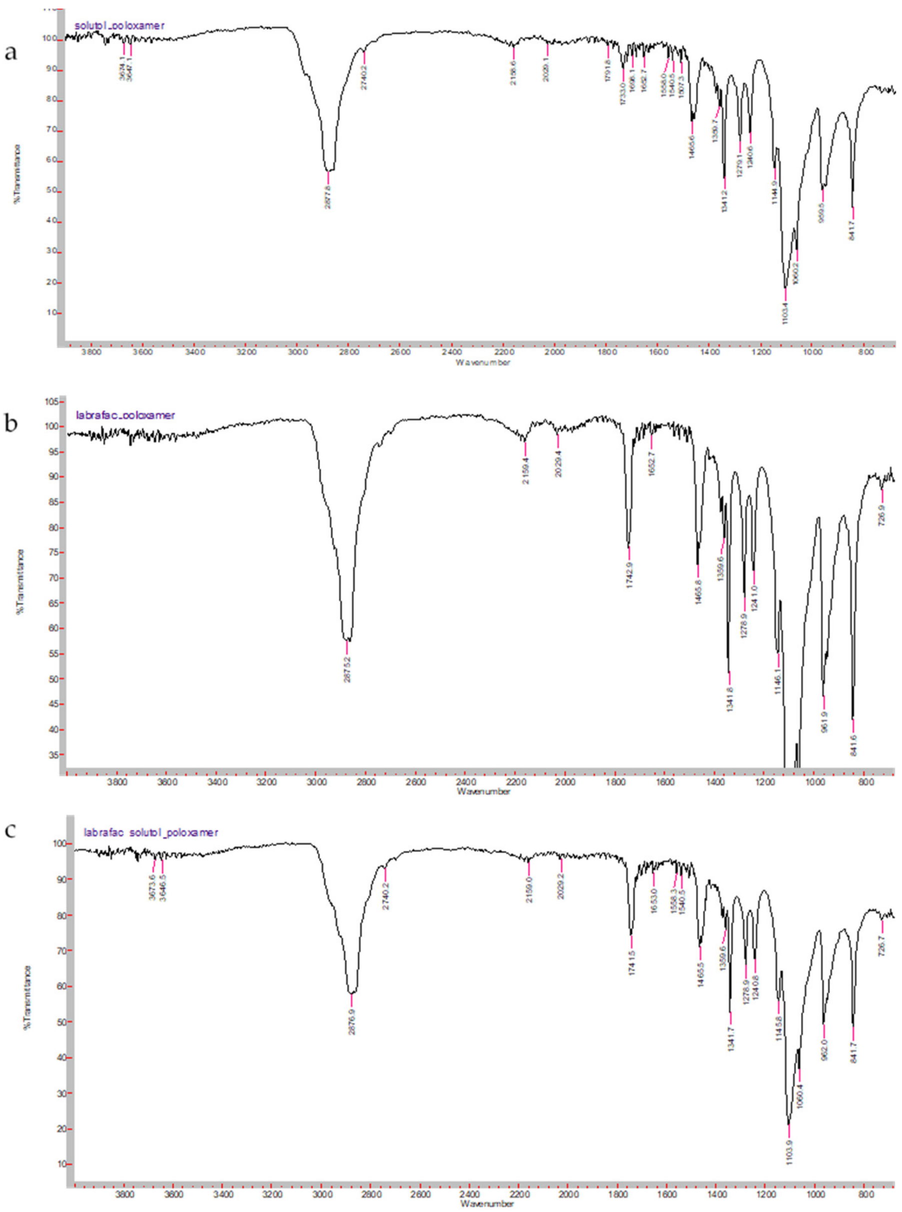

Figure S3. IR spectra of physical mixture of (a) kolliphor-poloxamer 407, (b) labrafac-poloxamer 407 and (c) kolliphor-labrafac-poloxamer 407.

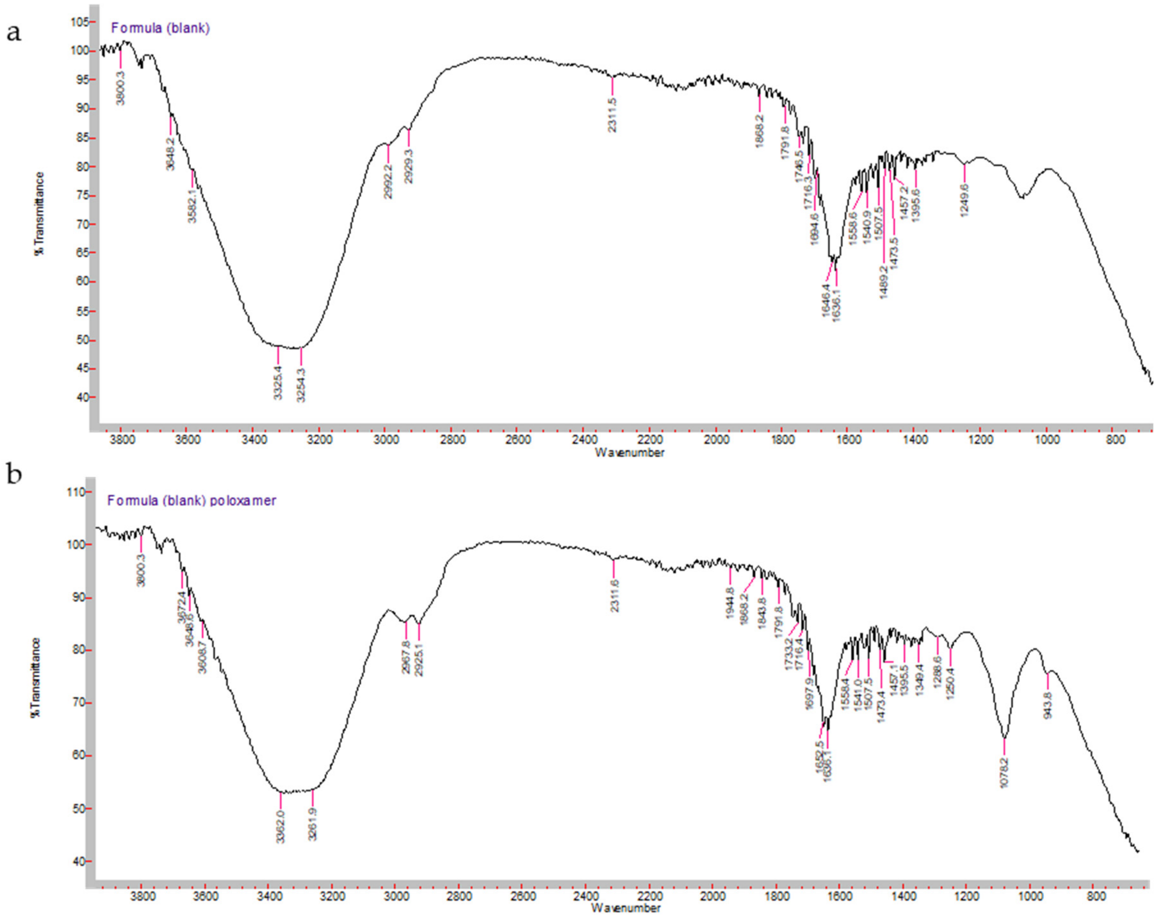

Figure S4. IR septra of (a) blank LNC and (b) blank LNC in P407 in situ gel.
